# A Low-Cost Paper Glucose Sensor with Molecularly Imprinted Polyaniline Electrode

**DOI:** 10.3390/s20041098

**Published:** 2020-02-17

**Authors:** Zheyuan Chen, Christopher Wright, Onder Dincel, Ting-Yen Chi, Jun Kameoka

**Affiliations:** 1 Department of Electrical and Computer Engineering, Texas A&M University, College Station, TX 77840, USA; zychen@tamu.edu (Z.C.); chriswright96@tamu.edu (C.W.); onderdincel@tamu.edu (O.D.); 2 Department of Materials Science and Engineering, Texas A&M University, College Station, TX 77840, USA; kevin0149@tamu.edu

**Keywords:** low cost, enzyme free, paper sensor, molecular imprinting, polyaniline, glucose sensing

## Abstract

For the hundreds of millions of worldwide diabetic patients, glucose test strips are the most important and commonly used tool for monitoring blood glucose levels. Commercial test strips use glucose oxidases as recognition agents, which increases the cost and reduces the durability of test strips. To lower the cost of glucose sensors, we developed a paper-based electrical sensor with molecularly imprinted glucose recognition sites and demonstrated the determination of various glucose concentrations in bovine blood solutions. The sensing electrode is integrated with molecular recognition sites in the conductive polymer. A calibration graph as a function of glucose concentration in aqueous solution was acquired and matched with a correlation coefficient of 0.989. We also demonstrated the determination of the added glucose concentrations ranging from 2.2 to 11.1 mM in bovine blood samples with a linear correlation coefficient of 0.984. This non-enzymatic glucose sensor has the potential to reduce the health care cost of test strips as well as make glucose sensor test strips more accessible to underserved communities.

## 1. Introduction

Diabetes mellitus is a chronic condition that affects millions of people around the world. The disease develops as a result of either disrupted insulin production (type I) or altered insulin absorption (type II) [1]. Diabetic patients must monitor their glucose concentration often to avoid complications, such as cardiovascular damage, nerve degeneration, and vision damage [2,3]. For this purpose, current glucose sensors utilize electrochemical glucose detection with glucose oxidase (GOx) to detect changes in glucose concentration and have been commercially available and commonly used for diabetic patients [4,5]. This enzyme-based method is costly and sensitive to pH and temperature. Furthermore, electrochemical detection of glucose based on GOx on a solid substrate is unreliable [6]. To improve such weakness, there have been multiple new glucose-sensing techniques, such as optical transducers [7,8], and electrical transducers [9,10,11,12] investigated; however, these approaches still require GOx structures to recognize glucose, although their reliabilities have been improved. As one of the most expensive diseases to treat, it is important to reduce the cost of detecting glucose levels as much as possible without sacrificing reliability and accuracy [13].

To address the need for easy, rapid and reliable glucose sensing with low cost, various non-enzymatic methods have been investigated and developed [14,15,16]. Among these methods, the molecularly imprinting (MIP) approach has attracted much attention. MIP is a promising process that works by the co-polymerization of functional monomers and cross-linking agents in the presence of a template molecule or its derivative [17]. The MIP approach has shown great advantages, such as its simple preparation, potential reusability, long-term durability, and stability as well as low-cost production. MIP technology has been applied in various fields, such as purification [18], chromatographic separation [19], chiral separation [20], and solid-phase extraction [21]. MIP has also been investigated in chemical/biosensors [17,22,23,24,25]. However, a simple, cost-efficient and easy-operating way to transduce specific signals has yet to be established. Polyaniline (PANI) is one of the conductive polymers that has been widely used as a sensing electrode due to its characteristics of stability, high conductivity, and easy synthesis. MIP-PANI can act as both the sensing electrode and the conductive electrode, with the additional advantage of being low cost. The electrochemical polymerization of aniline has been reported as one of the most common approaches for biosensing [26,27], which demonstrates good selectivity and sensitivity. However, electro-polymerization has some drawbacks. The method requires monomers that can be oxidized electrochemically and the substrate must be conductive but inert to react during the electro-polymerization process. Moreover, conducting polymers tend to detach from the electrodes after suffering mechanical stress, sonication, and sterilization [28,29,30]. Such drawbacks limit its potential application in disposable glucose test strips with long durability and stability.

In this paper, instead of GOx, we developed and demonstrated a low-cost paper glucose sensor by direct MIP technology. This process enables a simple one-step approach to the fabrication of PANI by polymerization of the aniline monomer with the template on paper strips, providing a glucose-sensing electrode and a signal-transducing electrode at the same time. The proposed device was fabricated by MIP in a solution to create a glucose-binding site integrated with a conductive PANI electrode. As a proof of concept, glucose concentration was determined in both aqueous and bovine blood solutions to evaluate the performance of the PANI paper sensors. This simple and straightforward approach makes the production of easy, rapid and robust glucose test strips possible and provides low-cost and reliable glucose-monitoring access to diabetes patients.

## 2. Materials and Methods

### 2.1. Molecularly Imprinted Polyaniline Paper Sensor Preparation

**Figure 1:**
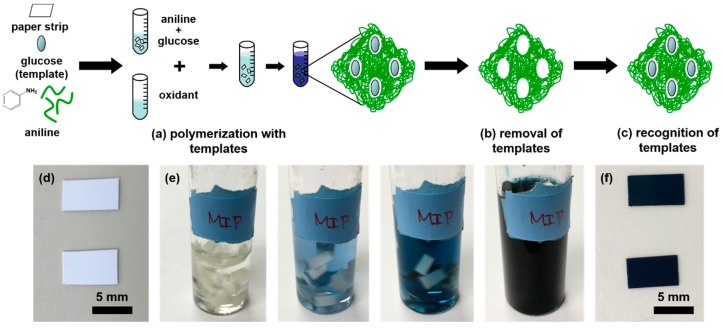
A schematic diagram of the molecular imprinting process for the fabrication of a paper sensor. The template represents glucose in this study. (**a**) Polymerization with templates. Glucose templates were mixed with aniline solution, the monomer, prior to the addition of the oxidant solution. Glucose-imprinted PANI, represented as the green regiment embedding templates in the schematic diagram, was then synthesized on the paper substrate. (**b**) Removal of glucose templates by washing the paper strips with deionized (DI) water. The cavities left in the polymer matrix create the specific recognition sites for the corresponding targets. (**c**) Recognition of templates on the paper sensor when conducting the glucose concentration measurement. (**d**) The polyester paper was cut into strips before polymerization. (**e**) The color of the polymerization solution changed from yellowish to light blue and finally became dark blue due to the doping process. The paper strips were completely immersed and were subject to vigorous stirring in the solution during polymerization. (**f**) The prepared paper strips with glucose imprinted PANI synthesized on both sides of the surface after the glucose removal process by washing with DI water.

A schematic diagram of the molecularly imprinting process for a paper sensor with glucose template is shown in Figure 1. The preparation process includes adding glucose molecules to aniline monomer solution and removing them once the polyaniline was synthesized.

The polyaniline was synthesized from an aniline monomer solution (99%, Sigma-Aldrich, USA) as shown in Figure 1a. 250 μL of the monomer solution was blended with 1 mL of 36.8% (*w*/*v*) hydrochloric acid (HCl, ACS grade, Macron, USA), which acts as the doping agent to increase the conductivity of PANI. An amount of 3 mL of DI water was added to the mixed solution to adjust the final concentration. Next, the template molecule, 50 mg of glucose (Sigma-Aldrich, USA), was blended into this solution to create glucose binding sites. To achieve the proper concentration for PANI polymerization, DI water was added until the total volume of the solution became 5 mL. For the final process, polyester paper substrates (iGage, USA) were cut into strips (7 × 4 mm, 250 μm in thickness) and dipped into the final solution to be soaked with the aniline-HCl solution, completely saturating the paper strips. The paper strips were soaked and stirred in the solution for at least 10 min before the oxidation process to ensure continuous saturation.

To prepare the oxidant solution, 0.609 mL of HCl was added into 4 mL DI water, followed by the addition of 409 mg of ammonium persulfate (APS, Sigma-Aldrich, USA). After 10 s of stirring, a bit more DI water was added to make the total volume of the prepared solution equal to 5 mL. HCl was also used to maintain the pH (pH = 0) and doping levels throughout the synthesis process.

Next, the synthesis process was initiated. While stirring the aniline solution, the oxidant solution was added drop by drop. The oxidant drops were dispensed in 5 s intervals from a pipette until the color of the solution changed from yellowish to dark blue (Figure 1e). The paper strips were kept immersed in the solution. To complete the synthesis process, the paper strips were removed from the solution, washed with DI water to remove excess PANI as well as glucose templates (Figure 1b), and then left out to dry for at least 8 h before usage.

### 2.2. Glucose Paper Sensor Fabrication

**Figure 2:**
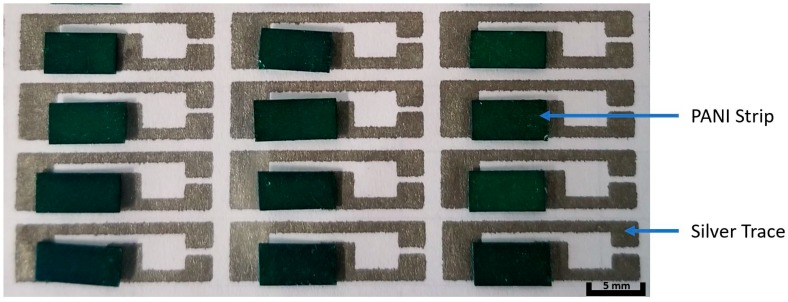
Photographic image of the paper sensor device after the initial silver traces were printed and before the secondary silver paste layer was formed to secure the strip to the device.

After the paper strips were fabricated (Figure 1f), the electrodes were printed by an inkjet printer using the Fujifilm Dimatix Material Printer 2830 with Novacentrix JS ADEV 291 ink. The operation parameters of the printer are listed in Appendix A. Silver traces were printed on a flat polyester film (Xerox, USA) with open space left for the PANI paper strips. The printed silver traces were cured at 110 °C for 1 h. Then, the PANI paper strips were fixed onto the open space and electrically connected to the silver traces via silver paste. Figure 2 shows the photographic image of the fabricated PANI paper sensor with glucose templates on the silver trace.

### 2.3. Fourier-Transform Infrared Spectroscopy (FTIR) Analysis

An Attenuated Total Reflectance-Fourier-transform Infrared (ATR-FTIR) Spectrometer was used to confirm the presence of glucose captured by the PANI paper sensor. The paper strips were exposed to 0 to 12 mM glucose, then rinsed with DI water after 5 min of the sample dispensing on the PANI surface. The paper strips were washed and dried in a vacuum overnight at room temperature. ATR-FTIR spectra of the surface were measured using the ATR module of the Thermo Nicolet 380 (Thermo, USA) FTIR spectrometer.

### 2.4. I-V Curve Measurement

**Figure 3:**
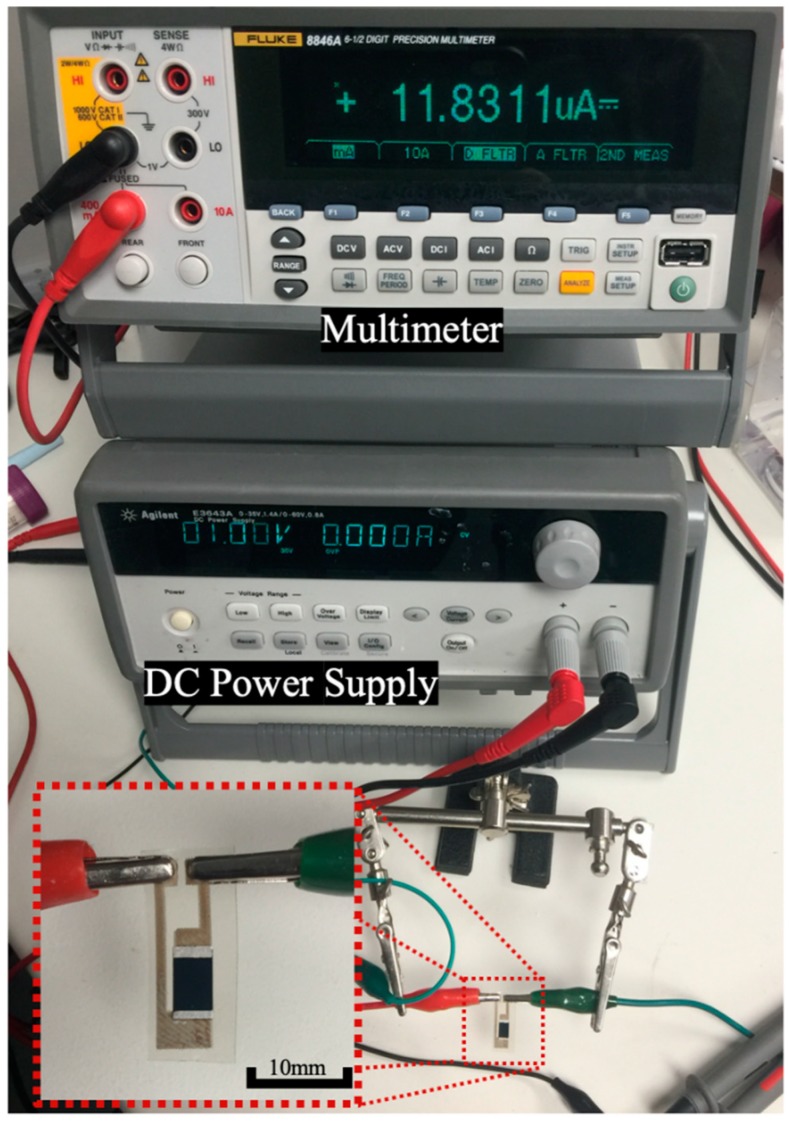
Photographic image of the experiment set-up for measuring the I-V curves of the paper sensor.

After the sensor fabrication, the I-V curve of the paper sensor was investigated. The experiment set up is shown in Figure 3. A multimeter (Fluke 8846A, USA) was used for recording the current. A DC power supply (Agilent E3643A, USA) was used for applying the voltage on the two ends of the silver trace. The voltage was applied from 0 to 10 V with a 1 V interval. The I-V curve measurement was conducted under air after dispensing DI water, and after dispensing 12 mM glucose in DI water. The sample volumes were the same (2 μL) and the measurement was conducted after 5 min of sample dispensing.

### 2.5. Glucose Concentration Measurement

The determination of glucose concentration was conducted by measuring the resistance change by connecting the two electrodes to the paper sensor. This approach is simple in operation and cost-efficient compared to other methods with an additional reference electrode or gate electrode. The resistance of the paper sensor was measured by a multimeter (Fluke 8846A) in a DC current mode. For glucose concentration determination in DI water, sample solutions with glucose concentrations from 0 mM to 12 mM were prepared. First, the direct current (DC) resistance of the device was measured as a reference level with DI water. An amount of 2 µL of the sample solutions with gradient glucose concentrations was dispensed on the surface of the PANI strips. All DC resistance was measured 5 min after sample dispensing. We compared the results based on the resistance change ratio (ΔR), which is the fraction of the sample with glucose solutions and the control samples. Equation (1) represents the calculation for ΔR:

ΔR = R_1_/R_0_, (1)

where R_1_ represents the measured resistance of the paper strip after the sample dispensing and R_0_ represents the original resistance before the sample dispensing.

For glucose concentration determination in bovine blood, a 250 mL bovine blood sample solution containing 35 mL of anticoagulant Citrate Phosphate Dextrose was used to characterize the performance of the paper sensor. The bovine blood was obtained from a healthy bovine from the Department of Veterinary in Texas A&M University. The blood test was conducted immediately after receiving the blood sample. The bovine blood solutions were added with 0, 0.1, 0.5, 2.2, 4.4, 6.7, 8.9, and 11.1 mM glucose as the sample solutions. The sample solutions were gently stirred before dispensing onto the PANI electrodes for glucose concentration detection. The volume of the blood samples was kept the same at 10 μL.

The resistance change of the paper sensor was detected before and after the sample solution dispensing. The resistance change ratios of the samples with different glucose concentrations added were calculated and normalized to the original resistance ratio of the control samples. The regression analysis was conducted by using software (Origin 2019 Pro). The regression analysis was also conducted with this software.

## 3. Results

### 3.1. FTIR Spectra

**Figure 4:**
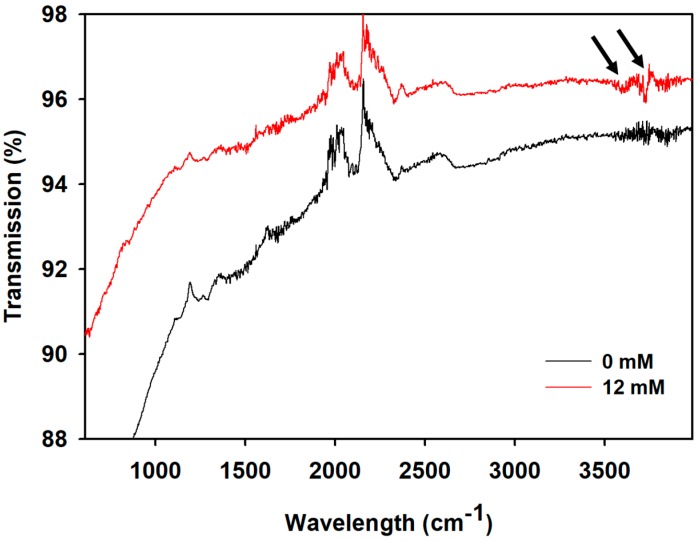
FTIR transmission result of the PANI paper strips after being dispensed with 0 mM (black line) and 12 mM (red line) of glucose in DI water solutions, respectively. The black arrows indicate the presence of an O-H bond that is contributed by glucose.

As shown in Figure 4, the peaks around 3500 to 3800 cm^−1^ in the FTIR spectra correspond to the presence of O-H bonds of glucose. The intensity of the peak of the paper strip with 12 mM glucose was significant compared to that with the 0 mM sample (as control). The noise presented in this range might be attributed to moisture in the air. The result indicate that the glucose was captured and recognized by the PANI paper strip.

### 3.2. I-V Curve of the MIP-PANI Paper Sensors

**Figure 5:**
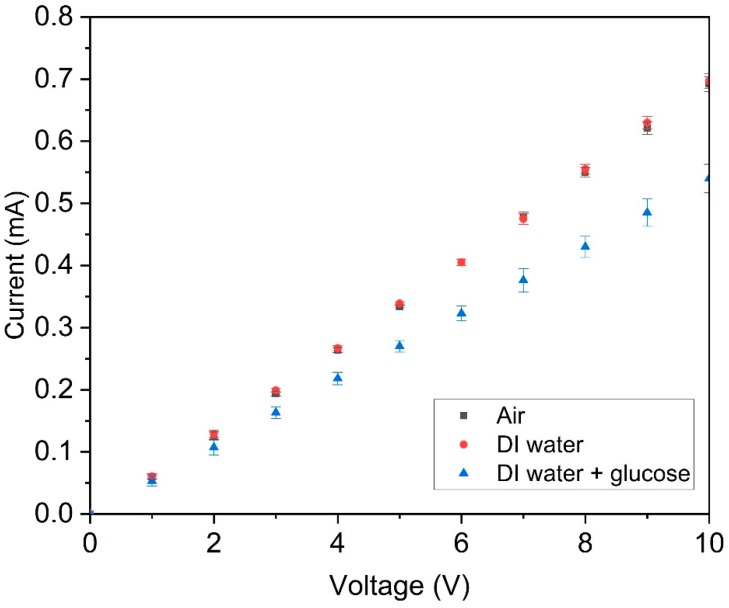
I-V curve of the PANI paper sensors. Each point represents an average of measurements of three identical PANI paper strips. The error bars represent the standard deviations of the three measurements. The black square, red circle and blue triangle represent the measurements under air, DI water and 12 mM of glucose in DI water.

The DC characteristics of the PANI paper sensors were investigated with gradient voltage increases from 0 to 10 V as shown in Figure 5. The results indicate that the PANI paper strips showed a linear and homogenous resistive response under uniformly increased DC voltage conditions with small standard deviations. A smaller current was observed in the paper sensor with the glucose in the DI water sample dispensed compared to those exposed in the air and with DI water only. A relatively large standard deviation was observed with the sensor dispensed with the glucose in the DI water sample.

### 3.3. Glucose Detection in Aqueous Solution

**Figure 6:**
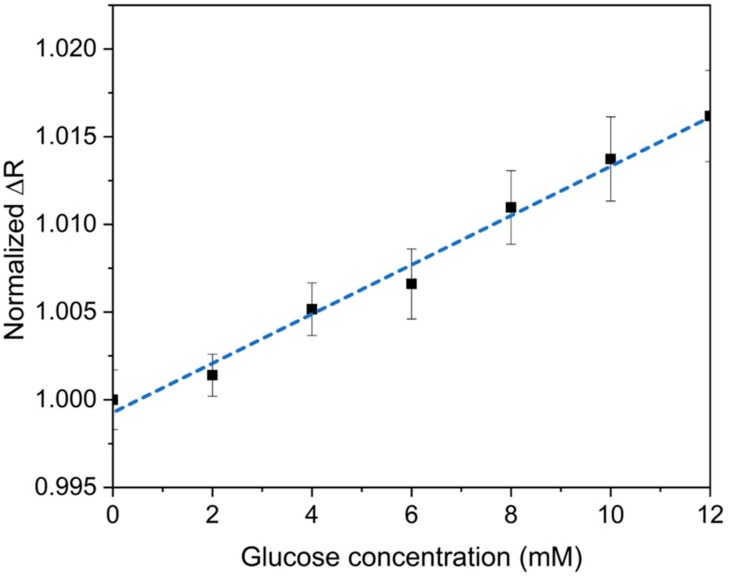
Graph of the normalized resistance change ratio as a function of glucose concentration in the DI water samples. The calibration curve (blue dash) of glucose concentration shows a linear correlation. Each point represents the average value of at least three identical tests in different paper sensors. The error bars are standard deviations calculated based on the resistance ratio measurements.

Samples with glucose concentrations from 0 to 12 mM were prepared for investigating the sensor performance. Figure 6 shows a graphical representation of the resistance as a function of glucose concentration. This graph indicates a clear linear correlation between the resistivity of the sensor and the concentration of glucose in the sample. The correlation coefficient was estimated to be 0.989 by the software using linear regression. The linear regression model was obtained as y = 0.0014x + 0.9993.

The limit of blank (LOB) of glucose in DI water samples is calculated based on Equation (2) [31]:

LOB = mean of blank + 1.645 (SD of blank) (2)

where mean of blank is the average value at blank sample and SD of blank is the standard deviation of blank sample. The limit of detection (LOD) of glucose in DI water samples is calculated based on Equation (3) [31]:

LOD = LOB + 1.645 (SD of low concentration)(3)

where SD of low concentration is the standard deviation at low concentration sample. Based on the calculation, the LOD for the paper sensor with DI water samples is estimated to be 1.0048 mM.

### 3.4. Glucose Detection in Bovine Blood

**Figure 7:**
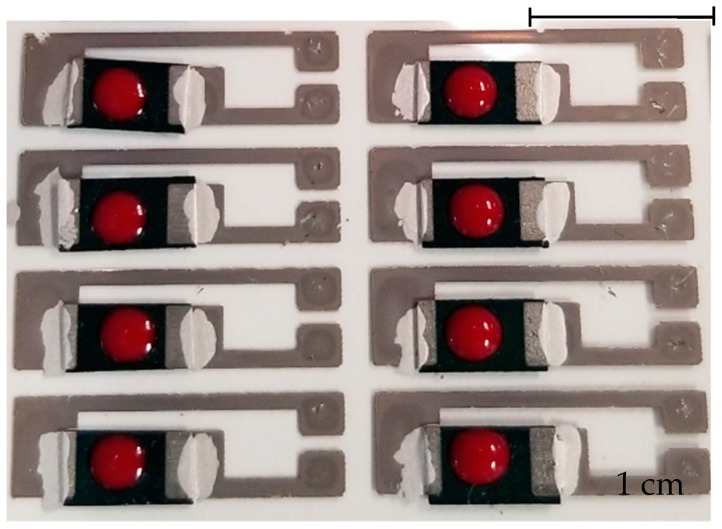
Photographic image of the paper sensor array devices after dispensing blood solution containing glucose.

Figure 7 shows the photographic image for paper sensors with the blood samples dispensed on the PANI electrodes.

As shown in Figure 6, the results for the determination of glucose concentration in the blood solutions indicated the linear increase of resistance changes as glucose concentrations increased. The result of this experiment also show that the normalized ratio of resistance change of the paper sensor was linearly correlated with the glucose concentrations ranging from 2.2 mM to 11.1 mM. However, the normalized resistance change was not significant due to low glucose concentrations of 0.1 and 0.5 mM. The glucose paper sensor showed a linear range of resistance ratio increase with a correlation coefficient of 0.984 with the added glucose concentrations ranging from 2.2 mM to 11.1 mM. In addition, the results show relatively large standard deviations compared to those in the DI water measurements.

The LOD of glucose detection in the blood samples was also studied by the same method mentioned in the DI water samples. Since the normalized resistance ratio at low glucose concentrations was not significant, the linear regression analysis was applied in the range of 2.2 to 11.1 mM of the added glucose. The linear model was obtained as y = 0.0651x + 0.8381. The LOD of the paper sensors in the blood samples was estimated to be 1.1713 mM.

## 4. Discussion

**Figure 8:**
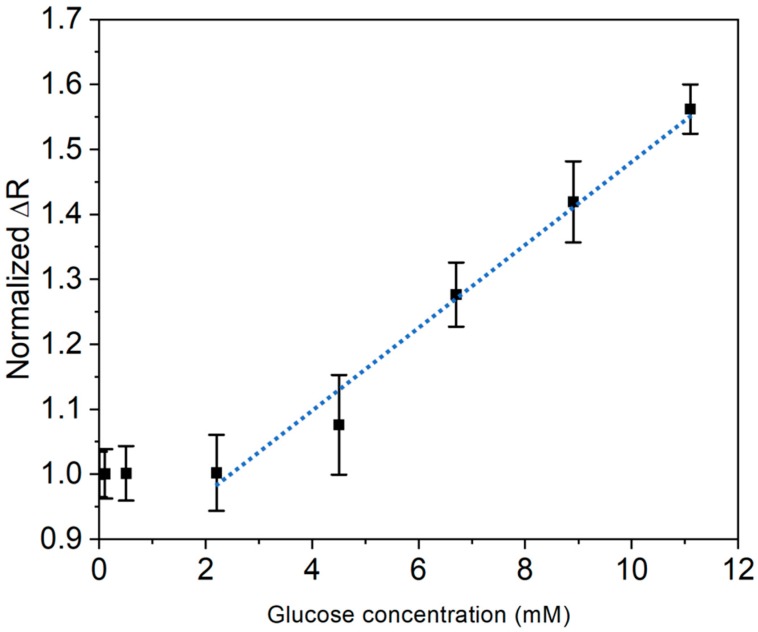
Graph of the normalized resistance change ratio as a function of the added glucose concentrastion in the bovine blood sample. The calibration curve (blue dash) of glucose concentration shows linear correlation ranging from 2.2 to 11.1 mM. Each point represents an average of at least three identical tests on different paper sensors. The error bars represent the standard deviations calculated based on the resistance ratio measurements.

The glucose detection mechanism can be simply explained by the change of resistivity if glucose binds to the MIP recognition sites on the PANI strips. For the detection of glucose in DI water (pH around 7), glucose captured on the PANI strips prevents holes from freely moving on the PANI surface. In this case, the decrease of the hole concentration in PANI results in the increase of the resistance as shown in Figure 6. With the same mechanism, the resistance increases linearly for a certain range of glucose concentrations as shown in Figure 8.

The I-V curve of the paper sensor with the glucose in the DI water sample dispensed showed reduced values compared to those exposed in the air and with DI water. The decrease of current indicated an increase in the resistance of the paper sensor at the given voltage. This is attributed to the binding of glucose when dispensing the glucose in DI water solution onto the paper sensor.

The normalized ΔR for each glucose concentration measured in blood samples presents much higher standard deviations than those measured in DI water. This is due to the complexity of blood samples dispensed on the polymer electrode. The glucose recognition sites could also be captured with molecules in the blood with similar size, shape, and charge as glucose, which might cause large standard deviations in glucose concentration determination. Additionally, the measurement was conducted 5 min after dispensing the sample solution, allowing more small molecules other than glucose to be captured on the polymer electrodes, which results in large deviations and a signal increase in the resistance measurement. In addition, blood cells or large biomolecules that tend to attach on the surface of the paper sensor might also affect the results.

The proposed PANI paper sensor is promising as disposable paper strips for glucose sensing. MIP-based glucose detection approach using expensive graphite/nanoparticle composites [22,24] requires multiple fabrication steps and increases the cost sharply. A gate-electrode by MIP method was also reported, but the additional electrode increases the cost of the sensor [25]. The proposed paper sensor provides a low-cost routine assay to diabetic patients with a moderate detection limit. Furthermore, the paper sensor is enzyme-free, which enables a long shelf-time with stability and durability when exposed to temperature and pH variations.

A limitation of the work could be the detection limit of the paper sensor, which hinders the application in very low-concentration situations. Furthermore, the reaction time is longer compared to traditional electrochemical glucose sensors in several seconds. Moreover, the dimensions of the paper strips could be optimized. Future work will be focused on improving the sensitivity of the paper sensor and the miniaturization of paper sensors which could further reduce the cost.

## 5. Conclusions

In this work, we developed and demonstrated a molecularly imprinted paper biosensor that has great potential as a low-cost alternative to determine blood glucose levels. The linear resistance response of the paper sensor strip can provide a low-cost, highly durable, and accurate glucose measurement method in blood. Other benefits of the paper-sensing strips include much lower temperature and humidity dependence that lead to a longer shelf life and ease of fabrication. On the contrary, GOx based sensors are more susceptible to temperature and pH fluctuations. The low-cost fabrication, accurate, and reproducible detection of paper sensor results can provide a new way of determining glucose concentration in the blood of diabetic patients.

## 6. Patents

We have filed the invention of paper sensor with patent application: YES. PCT/US2019/056303.

## Appendix A

**Table A1:** Summary of the settings used in the material printer for silver electrode printing.

Parameter	Nozzle Spacing	Nozzles Activated	Nozzle Diameter	Frequency	Cartridge Size
Value	254 µm	16	21 µm	30 kHz	10 pL
Parameter	Substrate Temperature	Jetting Frequency	Applied Voltage	Drop Spacing	Drop Angle
Value	30 °C	5 Hz	30 V	20 µm	4.4°
